# Complete chloroplast genome of *Prunus fasciculata* (Rosaceae), a species native to western North America

**DOI:** 10.1080/23802359.2021.1872439

**Published:** 2021-02-09

**Authors:** Yue-Ling Li, James Reed, Yu-Qian Xu, Da-Hao Qu, Zhong-Shuai Sun

**Affiliations:** a Zhejiang Provincial Key Laboratory of Plant Evolutionary Ecology and Conservation, Taizhou University, Taizhou, China; b California Botanic Garden, Claremont, CA, USA; c College of Life Sciences, Taizhou University, Taizhou, China

**Keywords:** *Prunus fasciculata*, *Prunus sensu lato*, chloroplast genome, phylogenomics

## Abstract

*Prunus fasciculata* is a wild species of *Prunus* native to western North America. Here, we reported the complete chloroplast (cp) genome of *P. fasciculata* (GenBank accession number: MW160273). The cp genome was 157,986 bp long, with a large single-copy (LSC) region of 86,068 bp and a small single-copy (SSC) region of 19,166 bp separated by a pair of inverted repeats (IRs) of 26,376 bp. It encodes 129 genes, including 84 protein-coding genes, 37 tRNA genes, and eight ribosomal RNA genes. We also reconstructed the phylogeny of *Prunus sensu lato* using maximum-likelihood (ML) method, including our data and previously reported cp genomes of related taxa. The phylogenetic analysis confirmed the sister group relationship between *P. fasciculata* and the remaining subg. *Prunus*.

Desert almond, *Prunus fasciculata* (Torrey) A. Gray, is an intricately branched, deciduous, stiff stemmed shrub in Rosaceae that is widely scattered in and around the Mojave Desert of western North America (Mason 1913; Rohrer 2014). *P. fasciculata* is the representative species of *Prunus* subg. *Prunus* sect. *Emplectocladus* (Mason 1913) which had been described as a distinct genus (Torrey 1851) and then treated as a section within *Prunus sensu lato* (Gray 1874). A number of recent phylogenetic studies of *Prunus sensu lato* based on molecular data (Bortiri et al. 2001; Lee and Wen 2001; Wen et al. 2008; Shi et al. 2013; Chin et al. 2014) supported that the American section *Emplectocladus* diverged the earliest within subg. *Prunus*. The subg. *Prunus* is economically very important, but circumscription of subg. *Prunus* and its taxonomic status in *Prunus sensu lato* have long been controversial due to the still unsolved phylogenetic system of *Prunus sensu lato* (Shi et al. 2013; Chin et al. 2014). By taking advantages of next-generation sequencing technologies, we can rapidly access the abundant chloroplast (cp) genomic data for phylogenetic research (Li et al. 2017; Liu et al. 2017). Therefore, we sequenced the whole cp genome of *P. fasciculata* to elucidate its phylogenetic relationship with other species in *Prunus sensu lato*.

Total genomic DNA was extracted from silica-dried leaves collected from California Botanic Garden (Claremont, CA) using a modified CTAB method (Doyle and Doyle 1987). A voucher specimen (CALBG_6525) was collected and deposited in the Herbarium of Taizhou University. DNA libraries preparation and pair-end reads sequencing were performed on the Illumina NovaSeq 6000 platform (San Diego, CA). The cp genome was assembled via NOVOPlasty (Dierckxsens et al. 2017), using the *Prunus rufa* cp genome (MN648456; Li et al. 2020) as a reference. Gene annotation was performed via the online program Dual Organellar Genome Annotator (DOGMA; Wyman et al. 2004). Geneious R11 (Biomatters Ltd., Auckland, New Zealand) was used for inspecting the cp genome structure.

The complete cp genome of *P. fasciculata* (GenBank accession number: MW160273) was 157,986 bp long consisting of a pair of inverted repeat regions (IRs with 26,376 bp) divided by two single-copy regions (large single-copy (LSC) with 86,068 bp; small single-copy (SSC) with 19,166 bp). The overall GC contents of the total length, LSC, SSC, and IR regions were 36.7%, 34.6%, 30.1%, and 42.6%, respectively. The genome contained a total of 129 genes, including 84 protein-coding genes, 37 tRNA genes, and eight rRNA genes.

We used a total of 24 additional complete cp genomes of the *Prunus sensu lato* species to clarify the phylogenetic position of *P. fasciculata*. *Prunus serotina* Ehrh. (NC036133) and *P. padus* L. (NC026982) in Subg. *Padus* were used as the outgroup. We reconstructed a phylogeny employing the GTR + G model and 1000 bootstrap replicates under the maximum-likelihood (ML) inference in RAxML-HPC v.8.2.10 on the CIPRES cluster (Miller et al. 2010). The ML tree (Figure 1) was consistent with the most recent phylogenetic study on *Prunus sensu lato* (Shi et al. 2013; Chin et al. 2014). The phylogenetic analysis confirmed the sister group relationship between *P. fasciculata* and the remaining subg. *Prunus*.

**Figure 1.**
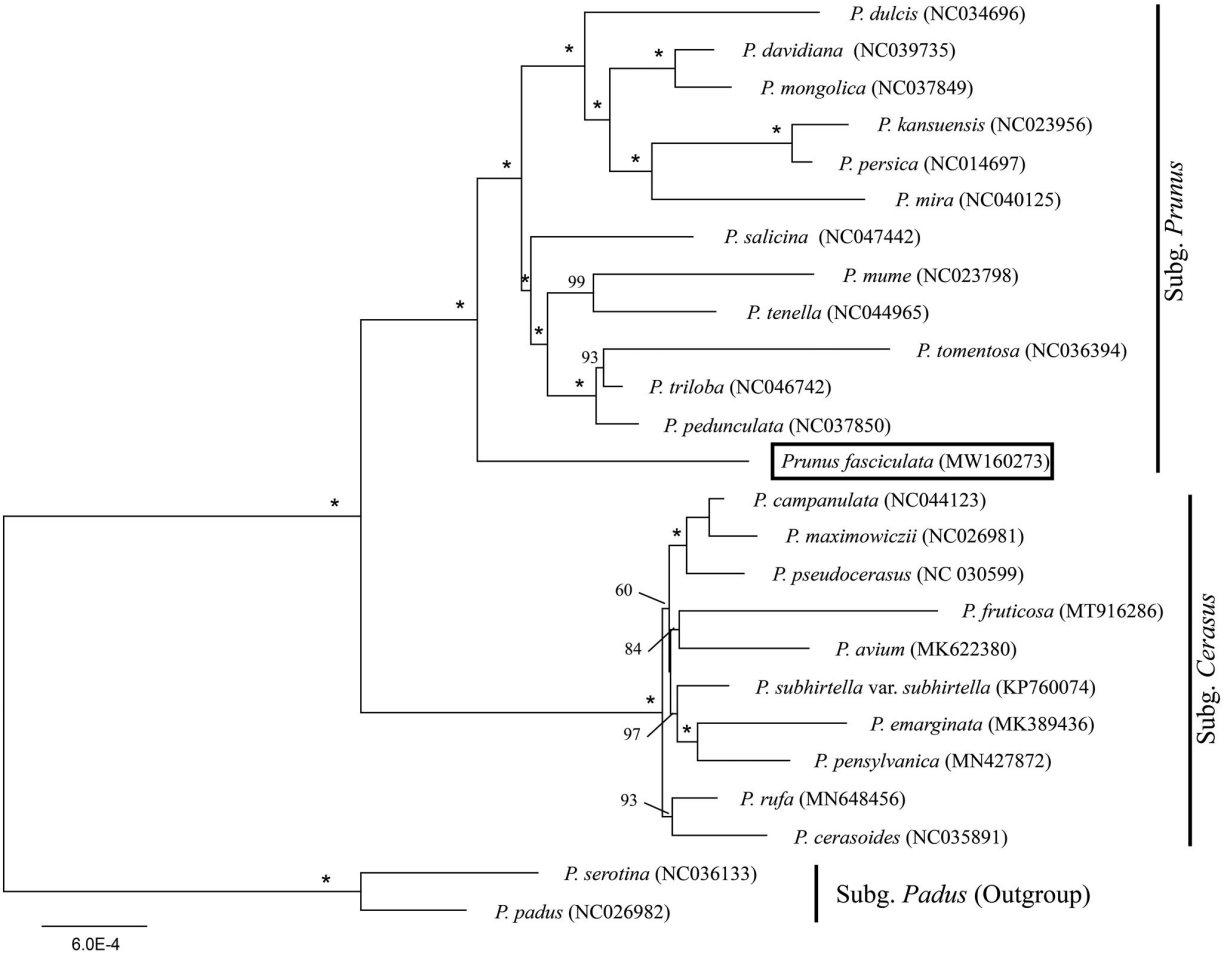
Phylogenetic tree reconstruction of 25 taxa of *Prunus sensu lato* using ML method. Relative branch lengths are indicated. Support values above the branches are ML bootstrap support; *100% support values.

## Data Availability

The genome sequence data that support the findings of this study are openly available in GenBank of NCBI at https://www.ncbi.nlm.nih.gov/ under the accession no. MW160273. The associated BioProject, SRA, and BioSample numbers are PRJNA677866, SRR13039950, and SAMN16774453, respectively.
